# Rotterdam Prostate Cancer Risk Calculator: Development and Usability Testing of the Mobile Phone App

**DOI:** 10.2196/cancer.6750

**Published:** 2017-01-06

**Authors:** Nuno Pereira-Azevedo, Luís Osório, Avelino Fraga, Monique J Roobol

**Affiliations:** ^1^ Department of Urology Erasmus University Medical Center Rotterdam Netherlands; ^2^ Urology Department Porto Hospital Centre Porto Portugal

**Keywords:** mHealth, prostate cancer, nomogram

## Abstract

**Background:**

The use of prostate cancer screening tools that take into account relevant prebiopsy information (ie, risk calculators) is recommended as a way of determining the risk of cancer and the subsequent need for a prostate biopsy. This has the potential to limit prostate cancer overdiagnosis and subsequent overtreatment. mHealth apps are gaining traction in urological practice and are used by both practitioners and patients for a variety of purposes.

**Objective:**

The impetus of the study was to design, develop, and assess a smartphone app for prostate cancer screening, based on the Rotterdam Prostate Cancer Risk Calculator (RPCRC).

**Methods:**

The results of the Rotterdam arm of the European Randomized Study of Screening for Prostate Cancer (ERSPC) study were used to elaborate several algorithms that allowed the risk of prostate cancer to be estimated. A step-by-step workflow was established to ensure that depending on the available clinical information the most complete risk model of the RPCRC was used. The user interface was designed and then the app was developed as a native app for iOS. The usability of the app was assessed using the Post-Study System Usability Questionnaire (PSSUQ) developed by IBM, in a group of 92 participants comprising urologists, general practitioners, and medical students.

**Results:**

A total of 11 questions were built into the app, and, depending on the answers, one of the different algorithms of the RPCRC could be used to predict the risk of prostate cancer and of clinically significant prostate cancer (Gleason score ≥7 and clinical stage >T2b). The system usefulness, information quality, and interface quality scores were high—92% (27.7/30), 87% (26.2/30), and 89% (13.4/15), respectively. No usability problems were identified.

**Conclusions:**

The RPCRC app is helpful in predicting the risk of prostate cancer and, even more importantly, clinically significant prostate cancer. Its algorithms have been externally validated before and the usability score shows the app’s interface is well designed. Further usability testing is required in different populations to verify these results and ensure that it is easy to use, to warrant a broad appeal, and to provide better patient care.

## Introduction

Prostate cancer is a serious health issue, accounting for 14% of all new cancers and 6% of total cancer deaths in men worldwide [1]. With the expected increase in life expectancy, the disease’s burden is projected to increase substantially [2]. However, neither the optimal balance between screening intensity and the risk of overdiagnosis (ie, detecting indolent disease) nor the ideal prostate cancer screening test or combination of tests have been determined [3].

To address these issues, screening trials were initiated. Recently, the third analysis of the European Randomized Study of Screening for Prostate Cancer (ERSPC), the world’s largest prostate cancer screening study, has been published. Currently, with more than 13 years of follow-up, the updated results show a stable relative benefit of screening (relative risk=0.79, ie, a 21% prostate cancer mortality reduction in favor of screening) but a still increasing absolute benefit [3]. The recently published findings show that to avoid one prostate cancer death, 781 men would need to be invited to screening and 27 additional prostate cancer cases will be diagnosed compared with no screening, both decreasing as compared with previous reports with shorter follow-up [3]. In summary, the number needed to screen and to treat to avoid one death from prostate cancer is decreasing and is now lower than the reported number needed to screen in trials for breast cancer [4].

Currently, the decision to perform a prostate biopsy is mostly based on the outcome of the serum prostate-specific antigen (PSA) test. However, the serum PSA level can increase in many situations, including benign (eg, benign prostatic hyperplasia) and inflammatory conditions (eg, acute prostatitis). Moreover, the optimal cutoff value has not yet been established [5].

Leveraging the decision of performing prostate biopsy solely on the PSA value, using a PSA value greater than 3.0 ng/mL as indication for Bx, resulted in 76% negative biopsy results [6]. Conversely, using a higher PSA threshold can neglect prostate cancer cases [7]. To address this lack of specificity, it is recommended that the PSA value should be combined with other relevant patient characteristics, using so-called risk calculators [2]. Even though many are available, currently it is not possible to provide a clear recommendation about which one to use in which situation (eg, first prostate biopsy, repeated prostate biopsy, patient with small prostate) because there are no direct head-to-head comparisons [8]. One scientifically sound and extensively validated risk calculator is the Rotterdam Prostate Cancer Risk Calculator (RPCRC), based on the ERSPC Rotterdam data [9].

**Figure 1:**
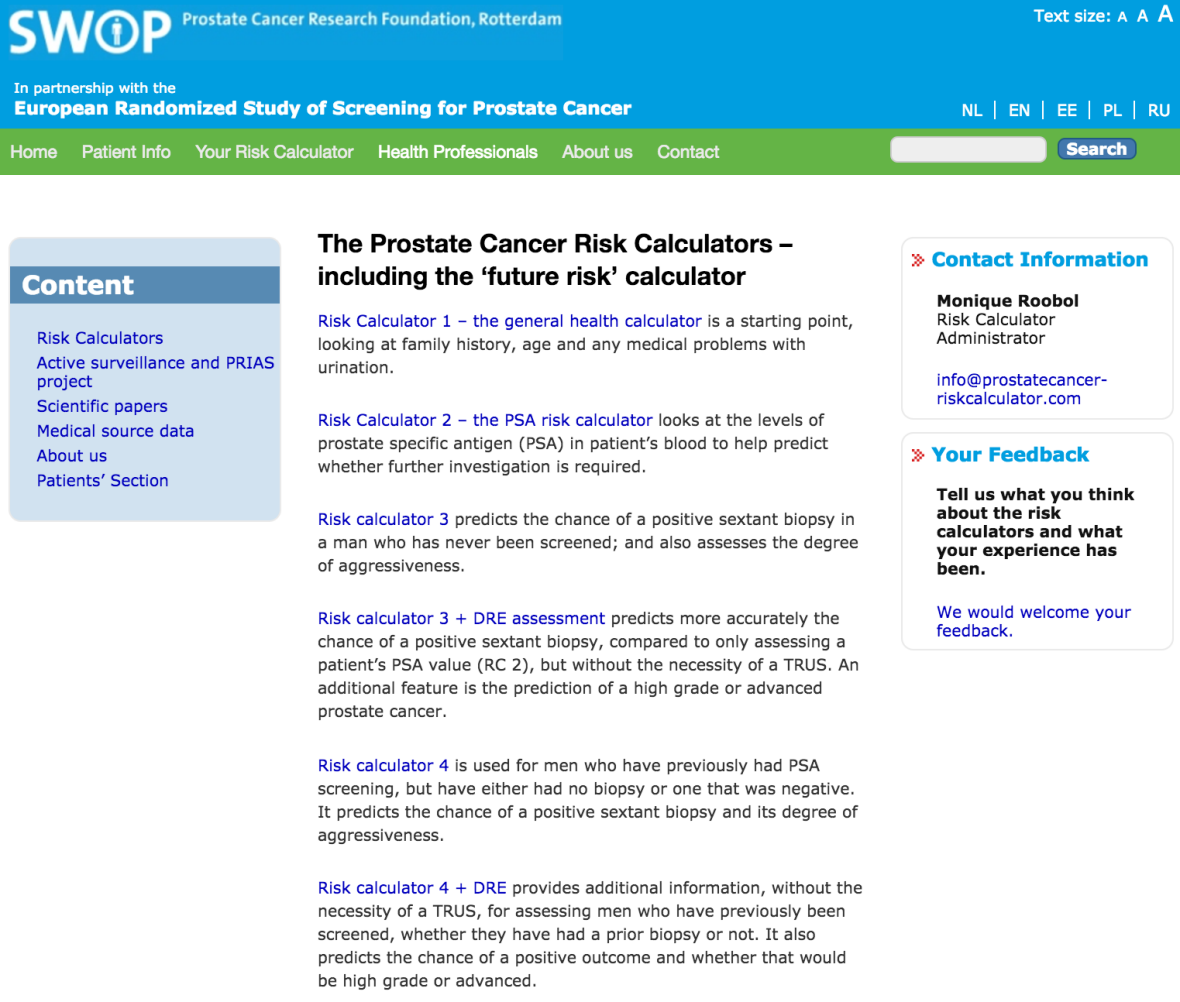
Screenshot of the Prostate Cancer Research Foundation website showing the prostate cancer risk calculators.

The RPCRC predicts the risk of a biopsy-detectable prostate cancer and also of potentially high-risk prostate cancer, defined as Gleason score ≥7 and clinical stage >T2b. This has important clinical implications as a way of decreasing overdiagnosis and overtreatment [3]. The different RPCRC algorithms provide an increasingly accurate risk estimation (ie, adding variables to the model increases its area under the curve, AUC). The algorithm uses information on PSA level, previous negative prostate biopsy, digital rectal examination (DRE) findings, prostate volume measurement, and transrectal ultrasonography (TRUS) findings. Additionally, the Prostate Health Index (phi), which aggregates the results from the Hybritech PSA, free PSA, and p2PSA (the [-2] form of proPSA), can also be used to further stratify prostate cancer risk [10]. All these different prediction models are available on the website of the Prostate Cancer Research Foundation (Figure 1) [11].

At present, mobile health (mHealth), the delivery of health care services via mobile communication devices, is a growing trend with more than 160,000 medical apps available, and the number is expected to grow even further, expedited by the ubiquitous presence of mobile phones and the continuous improvements in hardware and software [12,13]. To increase its usability and accessibility, the originally Web-based RPCRC [11] has been redesigned as an app, which has several benefits for the user.

Even though the app uses the same algorithms as the available Web-based risk calculators [11], the app’s proprietary step-by-step workflow ensures that, depending on the available information, the most complete algorithm is always used. In contrast, the website user has to initially choose a specific RPCRC, which may not be the most comprehensive available and inadvertently dismiss known clinical data.

Another strength of the app is that the calculations are performed in the user’s mobile phone (ie, it works offline), which ensures a safe user experience, bypassing issues with website blocking (eg, some facilities constrain Internet access) and with infrastructure and Internet service providers (eg, slow intranet or low-speed Internet access).

Several studies have shown that mHealth was well received by users, including health care professionals and patients, in both urban and rural settings. Some examples include the use of mobile phone–based guidance for rural health providers in Tamil Nadu, India [14], and the use of a gestational diabetes app by pregnant women in Oxford, United Kingdom [15]. Moreover, it has been documented not only in young adults [16], but also in older adults—both had a high degree of acceptance of apps that promoted physical activity [17].

The aim of this study was to design and develop a mobile phone app for prostate cancer screening, based on the RPCRC algorithms. Moreover, we sought to evaluate the usability of the developed app using IBM’s Post-Study System Usability Questionnaire (PSSUQ) [18].

## Methods

**Figure 2:**
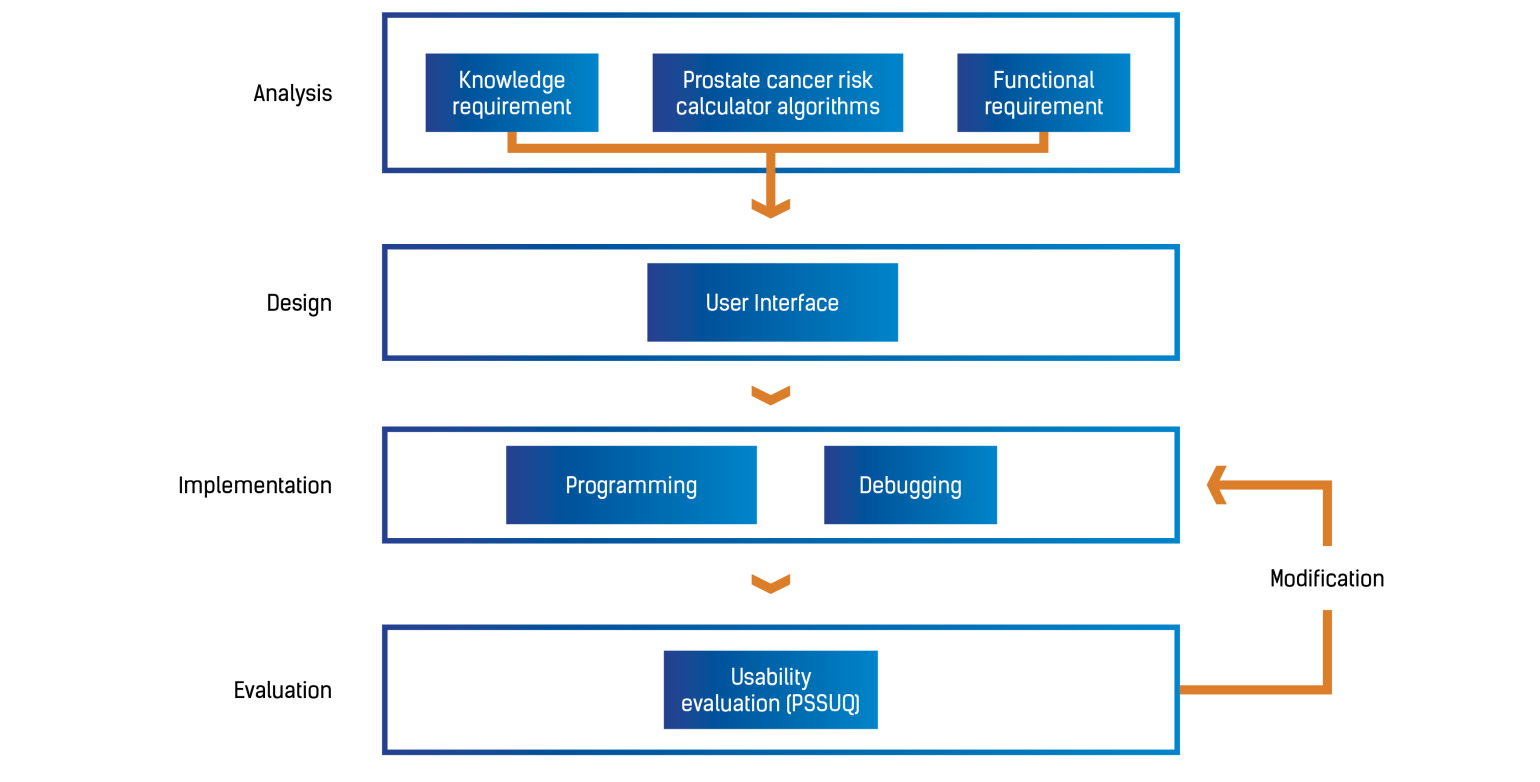
Study outline and research procedure. PSSUQ: Post-Study System Usability Questionnaire.

This study was structured according to the standard life cycle of system development: analysis, design, implementation, and evaluation, as shown in Figure 2.

### System Analysis

Knowledge and functional requirements for system implementation were assessed.

#### Knowledge Requirements

All risk calculator algorithms used in the app were developed based on the Rotterdam arm of the ERSPC, using the clinical data and prostate biopsy outcome from 3624 previously unscreened men and 2896 men with previous negative prostate biopsy. The following 4 models were built, with cumulative clinical information:

- Model 1—PSA alone;
- Model 2—PSA and DRE (normal/abnormal);
- Model 3—PSA, DRE (normal/abnormal) and DRE-assessed volume;
- Model 4—PSA, DRE (normal/abnormal), TRUS (normal/abnormal), and TRUS-assessed volume.

The predictive capability of the models within the RPCRC app were assessed in terms of discrimination (C statistic) for predicting the probability of both prostate cancer on biopsy and serious prostate cancer (defined as >T2b and Gleason score ≥7) [19]. Further details about the construction and the validation of the RPCRC algorithms have been previously published [19].

#### Functional Requirements

The system’s functional requirements were based on the available risk calculator algorithms that were developed by the Rotterdam ERSPC. To improve the RPCRC app usability, a unique decision tree was devised, with a multistep approach, to gather available clinical information: previous negative prostate biopsy, PSA value, DRE evaluation, TRUS evaluation, and phi value.

### System Design

The app’s user interface was designed to ensure the best possible experience, according to Apple’s design guidelines. The interface was based on the RPCRC decision tree, taking into account the clarity and ease of use, and was designed using the GNU Image Manipulation Program (GIMP).

### System Implementation

To ensure the best performance, a native iOS version was developed using Apple’s Xcode (Apple Inc), an integrated development environment that comprises a suite of software development tools, including debugging functions.

### System Usability Evaluation

Usability is defined as the measure of the ease with which a system can be learned and used, including its safety, effectiveness, and efficiency [20]. Usability is also a measure of the effectiveness of the interaction between humans and computer systems (ie, how do users perform tasks in the system) [21]. The usability of the RPCRC app was evaluated using IBM’s PSSUQ, which is currently in its third revision and consists of 3 domains: system usefulness, information quality, and interface quality [18]. These 3 domains cover 16 questions, rated on a Likert scale from 1 (I strongly disagree) to 5 (I strongly agree; Table 1). In addition, users also had the option to write their own comments. The PSSUQ was chosen because it is a popular usability testing instrument that was validated and showed discriminative validity, discerning applications with recognizably different quality [22]. Moreover, it has been used in several other mHealth studies [16,23-25].

Urologists, medical students, and general practitioners (GPs) were selected as end users; GPs were included because they are the first gatekeepers for prostate cancer screening, making the decision of whether or not to refer the patient to a urologist. Medical students’ evaluation is pertinent because they will be the urologists and GPs of tomorrow. An invitation to participate in the study was sent via email.

For the quantitative measurements (baseline characteristics, PSSUQ), means and standard deviations were calculated using software package IBM SPSS v20 (IBM Corporation).

## Results

### System Analysis

#### Knowledge Requirements

All risk calculator algorithms used in the app were developed based on the Rotterdam arm of the ERSPC, using the clinical data and prostate biopsy outcome from 3624 previously unscreened men and 2896 men with previous negative prostate biopsy [19].

In the original previously unscreened men, applying model 1 to model 4 resulted in AUCs from 0.69 to 0.79, respectively, for predicting prostate cancer and from 0.74 to 0.86, respectively, for predicting serious prostate cancer. In the previously screened group (men with at least one previous negative prostate biopsy), applying the same models, AUCs ranged from 0.62 to 0.69 for predicting prostate cancer and from 0.72 to 0.81 for predicting serious prostate cancer [19].

Several related papers that validate the algorithm of the RPCRC in different cohorts and compare the RPCRC with other calculators have been previously published, with good performance in the various settings [26-33].

#### Functional Requirements

**Figure 3:**
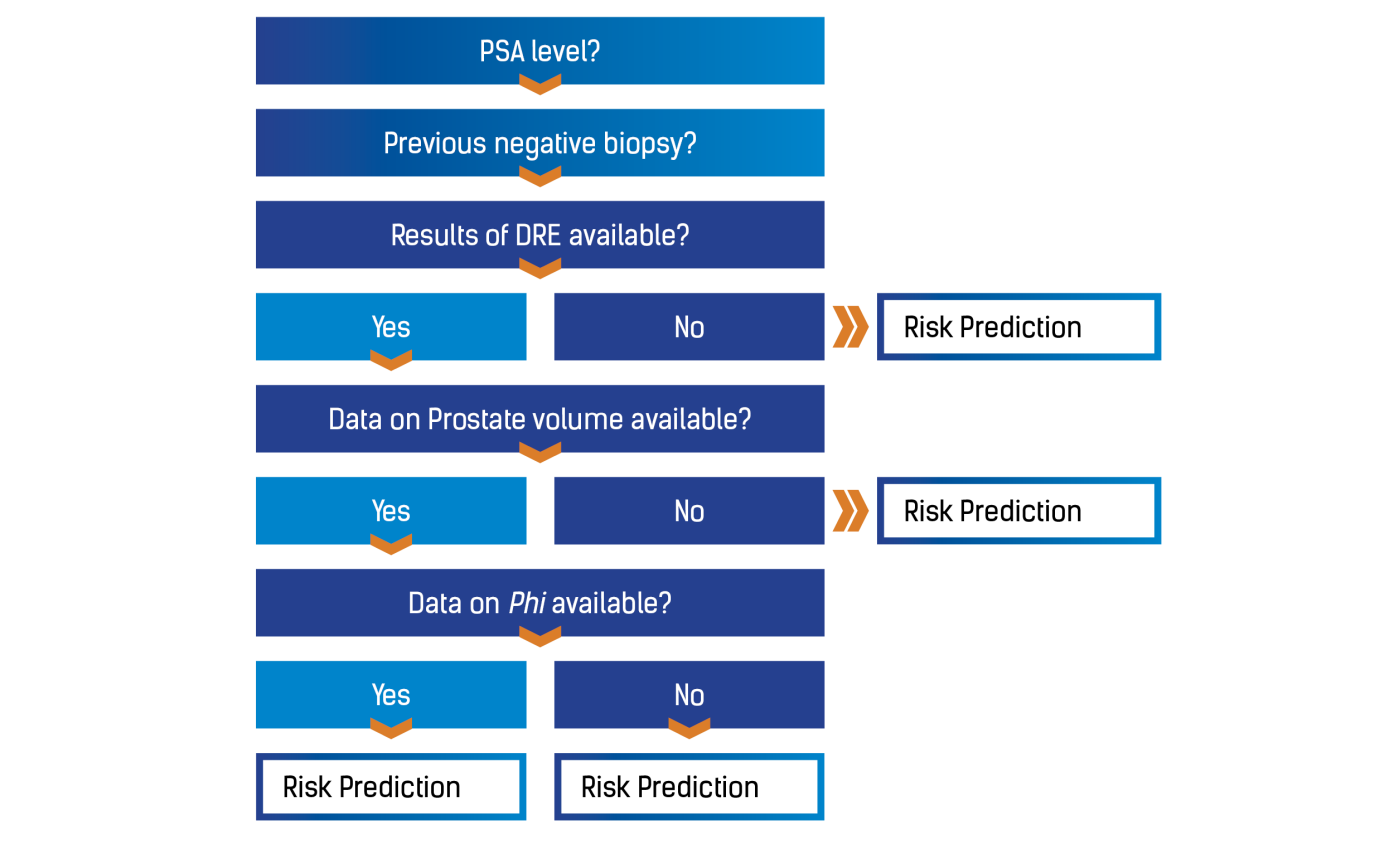
The Rotterdam Prostate Cancer Risk Calculator decision tree. PSA: prostate-specific antigen; DRE: digital rectal examination; phi: Prostate Health Index.

A unique decision tree was designed to ensure the app would always use the most powerful risk calculator model, depending on the available information (Figure 3). This ensures that the most significant available data is used in the most complete algorithm to compute with greater reliability the probability of a positive prostate biopsy and the risk of aggressive prostate cancer.

### System Design

**Figure 4:**
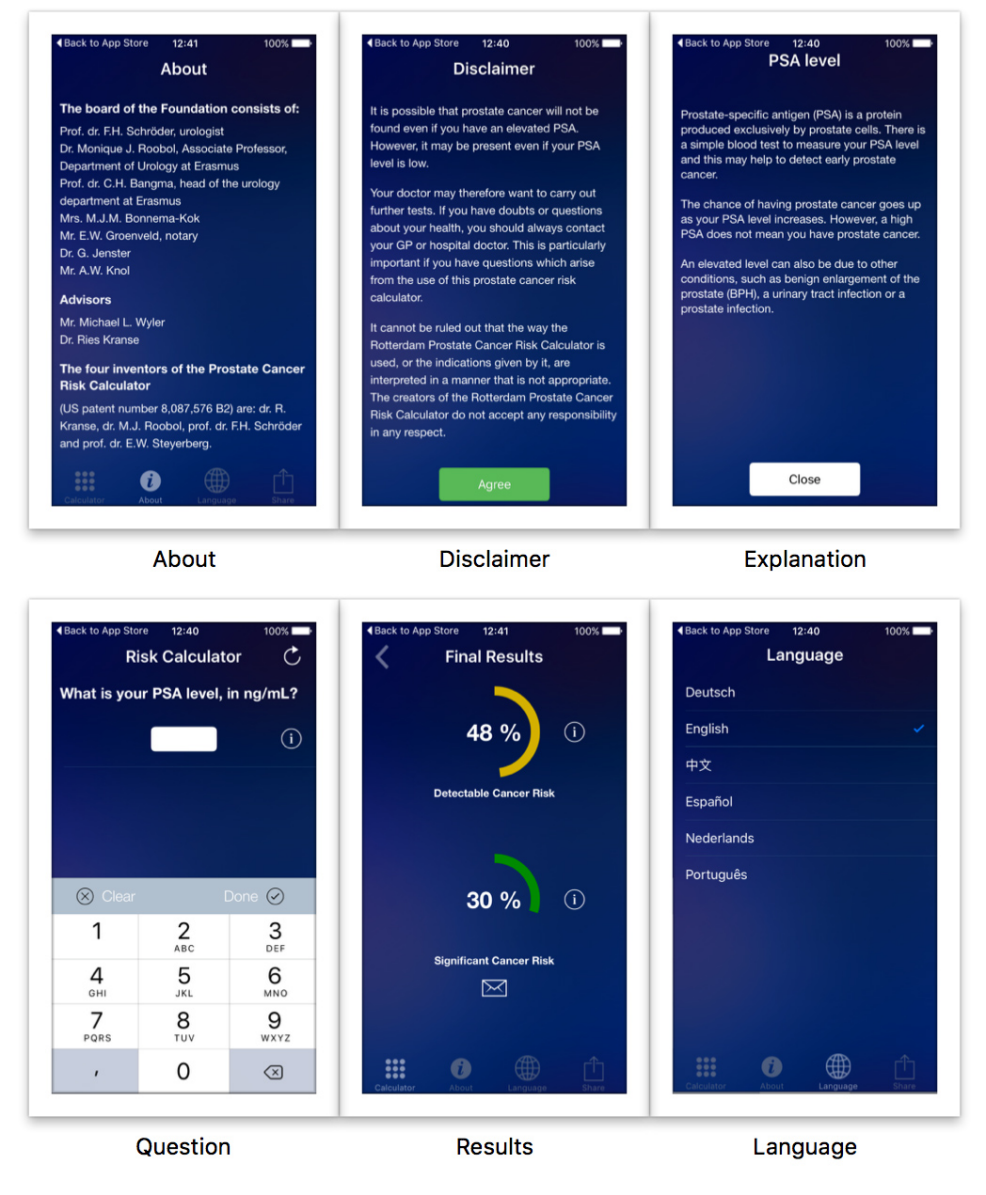
Screenshots of the Rotterdam Prostate Cancer Risk Calculator app, showing “About,” “Disclaimer,” “Explanation,” “Question,” “Results,” and “Language” screens.

The app design can be divided into 6 interface categories: disclaimer, question, explanation, language, results, and about (Figure 4). The disclaimer must be accepted by the user before using the app. A total of 11 questions were built into the app, and, depending on the answers, one of the different algorithms could be used to predict the risk of prostate cancer and of significant prostate cancer. All question interfaces are designed in a similar way. For every question, there is an interface with an explanation of the question. The results (ie, risk of prostate cancer and risk of aggressive prostate cancer) are shown in numerical (percentage) and graphic forms. The “about” screen details the scientific background of the risk calculators and lists all contributions. The user also has the option to choose the default language: Chinese, Dutch, English, German, Portuguese, and Spanish.

### System Implementation

The debugging of the app was performed within the Apple Xcode environment. All code errors were identified in a step-by-step approach, through the use of the intrinsic debugging tools, and were corrected according to Apple’s guidelines.

The functionalities of the app were assessed in various devices, namely, mobile phones and tablets, in the usability evaluation stage. Care was taken to ensure a consistent user experience across all devices.

### System Usability Evaluation

A total of 92 participants evaluated the usability of the app (response rate = 11%), among whom 28 (30%) were urologists, 29 (32%) were medical students, and 35 (38%) were GPs. The mean age of participants was 31 years and 62% were female. The calculated mean and standard deviation of the PSSUQ 16 questions are presented in Table 1. “It was simple to use this application” and “It was easy to learn to use this application” had the highest rating among the 16 items, with 4.80 out of 5 possible points.

The final scores of the 3 domains evaluated (ie, system usefulness, information quality, and interface quality) are presented in Table 2. The highest score (92%) was reported for system usefulness, and information quality got the lowest score (87%). These results show that the participants were, overall, satisfied with the usability of the app.

**Figure 5:**
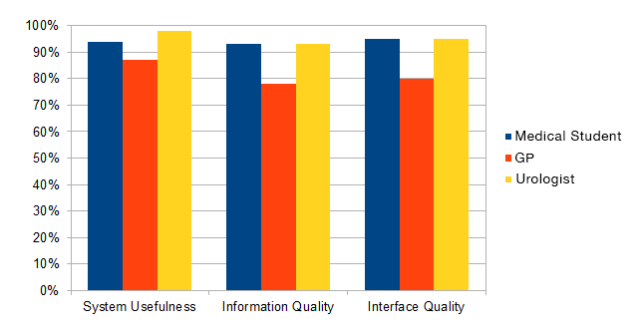
Percentage of actual score per item category and occupation of participants. GP: general practitioner.

Figure 5 shows the percentage of actual scores given by urologists, GPs, and medical students for system usefulness, information quality, and interface quality. The highest score was given for the system usefulness category by urologists.

**Table 1:** Means and standard deviations of the Post-Study System Usability Questionnaire result.

Category	No.	Item	Mean	SD
System usefulness	1	Overall, I am satisfied with how easy it is to use this application	4.67	0.557
	2	It was simple to use this application	4.80	0.399
	3	I was able to complete the tasks and scenarios quickly using this application	4.53	0.601
	4	I felt comfortable using this application	4.55	0.747
	5	It was easy to learn to use this application	4.80	0.426
	6	I believe I could become productive quickly using this application	4.34	0.905
Information quality	7	The application gave error messages that clearly told me how to fix problems	3.85	1.398
	8	Whenever I made a mistake using the application, I could recover easily and quickly	4.16	1.067
	9	The information (such as on-line help, on-screen messages and other documentation) provided with this application was clear	4.43	0.701
	10	It was easy to find the information I needed	4.47	0.654
	11	The information was effective in helping me complete the tasks and scenarios	4.52	0.673
	12	The organization of information on the application screens was clear	4.76	0.477
Interface quality	13	The interface of this application was pleasant	4.57	0.789
	14	I liked using the interface of this application	4.51	0.819
	15	This application has all the functions and capabilities I expected it to have	4.29	1.064
Overall	16	Overall, I am satisfied with this application	4.42	0.880
Total			4.48	0.832

**Table 2:** Scores per evaluation category of the Post-Study System Usability Questionnaire.

Item category	Actual score	Possible score	% Actual score
System usefulness	27.7	30	92
Information quality	26.2	30	87
Interface quality	13.4	15	89

## Discussion

### Principal Findings

Risk calculators are increasingly being used to stratify men at risk of prostate cancer. The RPCRC, previously only available digitally on the website [11], was based on the Rotterdam arm of the ERSPC, which started in 1993 in Europe to study the feasibility of population-based screening for prostate cancer and its effect on mortality [34]. This new app is publicly available on the Apple App Store [35].

To facilitate its use in clinical practice, we decided to create an mHealth version using the RPCRC algorithms. However, to simplify its use, a unique decision tree was created that offers a streamlined user experience, while incorporating additional information at every step. The app was well received by urologists and won the BJUI award for Best Urology App in 2015, presented at the American Urological Association Annual Meeting.

Starting with the total PSA value, a more complete assessment is built based on supplementary information regarding a previous negative prostate biopsy, DRE and TRUS findings, as well as phi value. Multiple external validations and comparisons of the RPCRC have shown that including more relevant information increases predictive capability [9].

This app builds on the ubiquitous presence of mobile phones to provide doctors and patients with a new way of using the RPCRC. Moreover, it maintains the ERSPC’s original goal to optimize prostate cancer screening, reducing unnecessary prostate biopsies and preventing the overtreatment of indolent prostate cancer while avoiding underdiagnosis. mHealth offers the opportunity to change the paradigm of health services, and prostate cancer, the second most common cancer worldwide, must be included in that effort [1].

In addition, it was designed and developed from day 1 by a multidisciplinary team, which included not only urologists but also other health care professionals, which has been shown to influence significantly the number of app downloads [36].

The strength of the RPCRC app is its development based on high-quality health information extracted from various published studies that validate the outcome of ERSPC risk calculator in multiple cohorts.

The IBM Computer Usability Satisfaction Questionnaire allowed the authors to obtain quantitative information regarding the app usability, which offered strong measures of usability. Moreover, taking into consideration that tests with only 5 participants are able to uncover 85% of usability issues, we believe most usability issues would be identified in this study, which included 92 users [37].

### Limitations

In this study, we only discuss the development of the iOS app, but further studies are under way to replicate this for other mobile platforms. Only medical students and health care professionals took part in the usability testing, which may represent a selection bias. In the near future, a similar evaluation will be done for patients.

### Conclusions

We created a scientifically valid and convenient mobile app for the RPCRC. The RPCRC has been designed to help patients and to assist health care professionals in the decision-making process. The app was found to be easy to use and, therefore, can be useful in the daily management of patients. The RPCRC app can be used in a clinical setting to better stratify the risk of prostate cancer, avoiding unnecessary biopsies and, consequently, reducing overdiagnosis and overtreatment.

## Abbreviations

AUC: area under the curve
DRE: digital rectal examination
ERSPC: European Randomized Study of Screening for Prostate Cancer
GP: general practitioner
p2PSA: (-2) form of proPSA
phi: Prostate Health Index
PSA: prostate-specific antigen
PSSUQ: Post-Study System Usability Questionnaire
RPCRC: Rotterdam Prostate Cancer Risk Calculator
TRUS: transrectal ultrasonography
